# Beyond non-adherence: qualitative insights into how patients strategically navigate pluralistic chronic care in West Africa

**DOI:** 10.1136/bmjopen-2026-123607

**Published:** 2026-10-15

**Authors:** Abdul-Basit Abdul-Samed, Ousseini Teoma Mamadou, Anicet Tounwendsida Kabore, Kezia Naa Amerley Akosua Amarteyfio, Salamatou Abdoul Karim Yamba, Aissa Diarra, Maurice Yaogo, Angela Owusu-Ansah, Martin McKee, Tolib Mirzoev, Irene Akua Agyepong, Lucy Yevoo, Neil Spicer, Augustina Koduah, Dina Balabanova

**Affiliations:** 1 Ghana College of Physicians and Surgeons, Accra, Ghana; 2 London School of Hygiene & Tropical Medicine, London, UK; 3 Laboratoire d’Etudes et de Recherche sur les Dynamiques Sociales et le Développement Local, Niamey, Niger; 4 Université Catholique de l'Afrique de l’Ouest - Unité Universitaire de Bobo-Dioulasso, Bobo-Dioulasso, Burkina Faso; 5 Ashesi University, Berekuso, Greater Accra Region, Ghana; 6 University of Ghana, Accra, Greater Accra Region, Ghana

**Keywords:** Delivery of Health Care, Integrated, Primary Health Care, PUBLIC HEALTH

## Abstract

**Abstract:**

**Background:**

Across West Africa, people living with chronic non-communicable diseases (NCDs) routinely seek care across biomedical, traditional and spiritual sectors. Patients often navigate these pluralistic health systems intentionally, responding to economic and social constraints, yet movement away from biomedical care is commonly interpreted as non-adherence. We examine how and why patients in West Africa negotiate their NCD care pathways across multiple sectors, and how they exercise agency within these constraints.

**Methods:**

We used a multiple qualitative case design, drawing on data from 11 purposively selected municipalities and districts in Burkina Faso, Ghana and Niger. Semistructured interviews were conducted with 84 participants: people with hypertension, diabetes or common mental health conditions (n=29); biomedical providers (n=29); traditional and spiritual healers (n=13); caregivers (n=3) and policy makers (n=10). Interviews in English, French and five local languages were analysed thematically, guided by frameworks on healthcare pluralism, patient agency and care-seeking trajectories.

**Results:**

Three patterns of therapeutic navigation emerged across all three countries, shaped by collective decision-making within extended family and peer networks, economic constraints and expectations of cure. First, when biomedical costs became untenable, patients shifted from biomedicine to traditional providers, carrying their diagnoses into the folk sector and often returning to biomedical care when resources allowed. Second, some patients concurrently maintained biomedical treatment while running self-managed traditional regimens, assigning distinct functions to each sector: biomedicine for diagnosis and monitoring, traditional medicine for the prospect of cure. Third, for mental health, families followed a spiritual-first trajectory from the outset, driven by explanatory models of spiritual causation rather than service availability or cost. Across all three patterns, patients frequently concealed their pluralistic practices from biomedical providers, fearing reprimand or finding no clinical space in which to disclose.

**Conclusion:**

Pluralistic care-seeking is a form of deliberate, conditioned agency, not simple non-adherence. Improving chronic NCD care requires more sustainably affordable biomedical services, consultation environments that enable open disclosure of pluralistic practice without penalty and health indicators that capture therapeutic navigation rather than biomedical endpoints alone.

**STRENGTHS AND LIMITATIONS OF THIS STUDY**

- This multicountry qualitative study drew on 84 semistructured interviews across Burkina Faso, Ghana and Niger, purposively sampling patients, biomedical providers, traditional and spiritual healers, caregivers and policy makers to allow comparison across roles and settings.
- Interviews were conducted in seven languages, with translation and bilingual checking of technical and culture-specific terms, though some loss of nuance in translation is possible.
- Analysis was abductive and theory-informed, combining Kleinman’s healthcare-sectors model, a theory of agency and the therapeutic itineraries framework.
- The design was largely cross-sectional, so care trajectories were reconstructed from participants’ retrospective accounts rather than observed prospectively.

## Introduction

 Across West Africa, chronic non-communicable diseases (NCDs) are rising faster than health systems can respond. Nearly half a million deaths were attributed to hypertension in 2019,^1^ and estimated treatment discontinuation rates reach 42.7% for hypertension and 54.4% for diabetes in sub-Saharan Africa.^2 3^ These patterns are often labelled as non-adherence, implying failure to follow care. However, such framing obscures how patients actively strategise their care within social and health system constraints.

Discontinuation of biomedical treatment does not always imply abandonment of care. Many patients navigate multiple therapeutic pathways, combining biomedical, traditional and spiritual care in ways largely overlooked by NCD frameworks focused on biomedical adherence.^4–7^ In Burkina Faso, Ghana and Niger, 60–80% of populations use traditional medicine as a primary healthcare resource (online supplementary table s1). The use of traditional, complementary and integrative medicine (TCIM) is therefore a sustained and integral part of healthcare systems rather than a residual practice.

Lay explanatory models (EMs) commonly combine dietary and bodily understandings with beliefs about spiritual causation, curses and spirit attacks. Patients often seek biomedical diagnosis while maintaining traditional interpretations of causation and cure.^8 9^ Thus, movement away from biomedicine does not reflect ignorance of its value. For mental health conditions, spiritual causation is frequently the dominant explanatory framework from the outset, shaping care-seeking before considerations of service availability arise.^10^

Global and regional strategies increasingly recognise TCIM as part of universal health coverage.^6 11 12^ The WHO Global Traditional Medicine Strategy 2025–2034 calls for stronger evidence, regulation and integration of safe, effective TCIM into national health systems.^11^ Several West African countries have adopted TCIM policies, with countries like Ghana going further by co-locating traditional medicine within public facilities at scale.^13–15^ However, policy development has outpaced evidence-based practice. To our knowledge, no comparative, multicountry qualitative study has examined how patients in West Africa navigate NCD care across biomedical and TCIM sectors. We address this gap by analysing what drives care decisions, how these vary across conditions and whether policy contexts shape patient experiences.

Drawing on Emirbayer and Mische’s concept of agency as purposeful action within structural constraints,^16^ we view agency as a collective, adaptive process rather than individual choice. We examine how patients and families in Burkina Faso, Ghana and Niger develop and adjust care strategies across biomedical, traditional and spiritual sectors for hypertension, diabetes and mental health, and what these reveal about the requirements for safer, more effective chronic care in pluralistic health systems.

### Study settings

This study uses differences in TCIM integration in Burkina Faso, Ghana and Niger as a comparative lens within broadly similar contexts of medical pluralism and NCD burden. The countries share key sociocultural features shaping healthcare-seeking, including extended family decision-making and coexistence of biomedical and traditional care, but differ in economic development, insurance coverage and formal TCIM integration.^17–19^ Ghana has a higher GDP per capita, wider insurance coverage (~56%) and the most extensive integration of traditional medicine, including herbal units in about 55 public facilities.^14 15^ Burkina Faso has an established but less institutionalised framework, with insurance coverage below 10%.^20 21^ Niger has a comprehensive regulatory framework on paper, including a Ministry of Health directorate, but limited operational integration; insurance coverage is below 2%, and out-of-pocket spending is among the highest in the region^22^ . Comparative indicators for the three countriees, and sources from which they are drawn, are presented in Table S1 (online supplementary table s1).^23–25 26 27 28 29 30 31 32 33 34 35–39^

## Methodology

### Study design

We employed a multiple qualitative case study design, drawing on data from the NIHR Global Health Research Centre for Non-Communicable Disease Control in West Africa (STOP-NCD), a 5-year research consortium operating in Burkina Faso, Ghana and Niger.^40^ Data were collected across sites established within the STOP-NCD infrastructure. In Ghana, Ledzokuku Municipality was purposively included because Lekma Hospital hosts a formally integrated herbal medicine unit, enabling direct observation of experiences within an integrated facility.

### Conceptual framework

Three interconnected frameworks guided the analysis. Kleinman’s healthcare systems model distinguishes popular, folk and professional sectors and uses EMs to separate biomedical disease from lived illness.^41^ Emirbayer and Mische’s theory of agency, as temporally embedded and socially situated, frames how patients evaluate options and project future outcomes within structural constraints.^16^ The therapeutic itineraries framework, drawing on Mendoza *et al*, conceptualises care-seeking as a fluid, adaptive journey shaped by evolving symptoms, economic circumstances, family dynamics and illness beliefs.^4^

Together, these frameworks allow examination of both observable care trajectories and the interpretive, social processes through which they are constructed and adjusted over time (figure 1). We use ‘therapeutic navigation’ to describe patterned movements across biomedical, traditional and spiritual sectors over time; ‘conditioned agency’ to describe purposeful action shaped by structural conditions; and ‘concealment’ to describe a relational outcome of clinical and institutional environments in which patients withhold information about pluralistic care-seeking.

**Figure 1:**
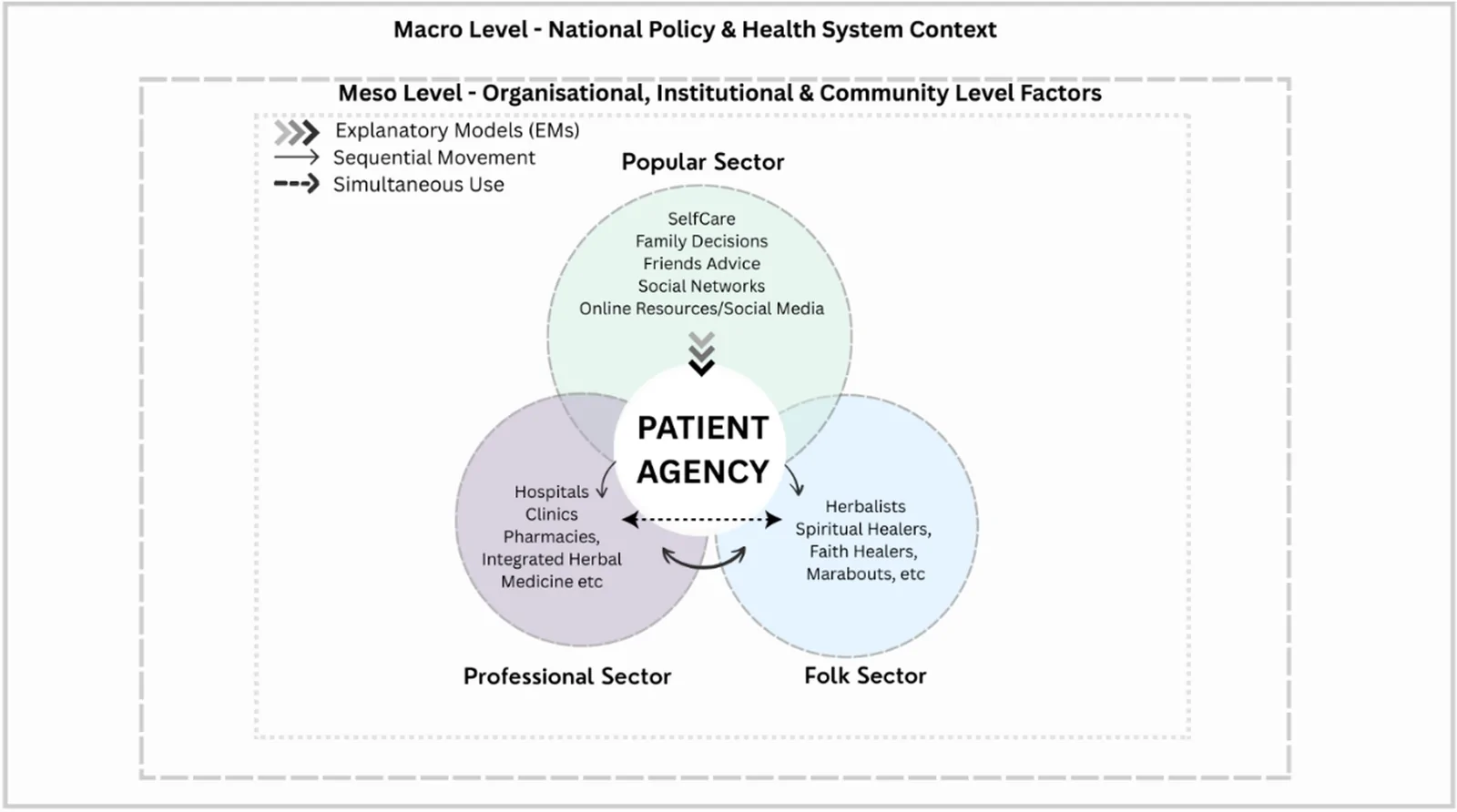
Conceptual framework—overlapping domains of care. Our conceptualisation is that care-seeking encompasses the three overlapping sectors, with a range of structural conditions that shape movement between them across meso and macro levels, and patient agency at the centre, operating across sector boundaries within these conditions. The arrows illustrate the bidirectional navigation between sectors, and the nested frames represent health system and societal determinants.

### Sampling and data collection

The study used purposive sampling to capture diverse perspectives from facility and community settings. Participants included people living with hypertension, diabetes or mental health conditions (n=29); biomedical providers, including physicians, nurses and medical herbalists (n=29); traditional and spiritual healers (n=13); family members and caregivers (n=3) and policy makers (n=10) (table 1). In each country, data were collected from a mix of urban and rural settings selected in consultation with country teams to reflect variation in service availability and TCIM use (table 1).

**Table 1:** Summary of study participants and study sites

Participant type	Ghana (n)	Burkina Faso (n)	Niger (n)	Total (n)
Patients	9	8	12	29
Biomedical/care providers (including medical herbalists)	6	14	9	29
Traditional/spiritual healers	2	6	5	13
Family members/caregivers	0	3	0	3
National-level policy makers	2	8	0	10
Total interviews analysed	19	39	26	84
Study sites	Ga South and Ledzokuku municipalities in the Greater Accra Region	Dô and Dandé in the Hauts-Bassins Region, and Koudougou and Réo in the Centre-Ouest Region	Communal Arrondissement 5 in Niamey; Tahoua Commune and Madaoua in the Tahoua Region; and 17 Portes and Mayahi in the Maradi Region	

Each country team developed and adapted its own semistructured topic guides, in the relevant languages, around a common set of domains agreed across the consortium and informed by community engagement (see Patient and public involvement). Common domains covered illness narratives and beliefs about causation; care-seeking trajectories and movement between biomedical, traditional and spiritual sectors; family and social influences on decision-making; economic barriers and perceptions of the efficacy and safety of each type of care. Within each country, role-specific versions were used: guides for patients and caregivers foregrounded illness experience, decision-making and cost; guides for biomedical providers and for traditional and spiritual healers focused on their interactions with patients using other sectors, disclosure and perceived risks; and guides for policy makers addressed governance, integration and system responses. Developing and adapting guides locally preserved linguistic, cultural and role-appropriate framing across settings, while the shared domains and common analytical frameworks maintained comparability across sites at the analysis stage. Interviews lasted 30–90 min, were audio-recorded with consent and were conducted in English, French, Twi, Hausa, Zarma, Mooré and Dioula. Interviews conducted in local languages were transcribed and translated into French or English by trained research assistants; French-language transcripts were subsequently translated into English using DeepL^42^ and checked by bilingual team members for accuracy of technical and culture-specific terms.

### Analysis

We used abductive thematic analysis. The three frameworks were applied to structure the analysis rather than to design the data collection instruments, and each played a distinct but complementary role. Kleinman’s healthcare-sectors model provided the initial deductive coding frame, distinguishing movement between popular, folk and professional sectors and separating biomedical disease from lived illness through EMs. The therapeutic itineraries framework structured the longitudinal reading of the data, allowing us to trace how care-seeking unfolded and was adjusted over time and to identify the recurring patterns of navigation. Emirbayer and Mische’s theory of agency served as the interpretive lens, used to explain why patients and families moved between sectors as they did—how they drew on past experience, projected future outcomes and made practical evaluations under constraint. Coding was conducted in NVivo 12, moving iteratively between data and frameworks to generate analytical categories; this common analytical structure was applied across the three country datasets to preserve comparability despite locally developed, role-specific topic guides.^43 44^ Saturation was reached when the three navigation patterns achieved internal coherence across conditions and country settings.^45^

All themes were analysed across participant strata and countries; patient and family accounts were systematically compared with those of biomedical providers, traditional and spiritual healers and policy makers to triangulate interpretations and to identify points of convergence and divergence. Reporting followed the Consolidated Criteria for Reporting Qualitative Research (COREQ) 32-item checklist (online supplementary table s2).

### Patient and public involvement

Community engagement through the STOP-NCD consortium informed the development of the research questions and interview topic guides. Patients were not involved in the data analysis. Findings will be disseminated through country teams and partner organisations in each setting, liaising with a network of stakeholders that includes patient representatives.

## Results

Consistent themes emerged across the 84 interviews in Burkina Faso, Ghana and Niger. Three patterns of therapeutic navigation were identified in which patients and families exercised deliberate, purposeful agency within structural constraints; concealment recurs across all three as a relational response to clinical environments rather than a strategy in its own right. Each pattern reflects a distinct configuration of constraints and decision-making logic, and some patients exhibited more than one pattern across their illness journeys as circumstances shifted. Hypertension and diabetes generated broadly similar trajectories, while mental health diverged fundamentally. Before presenting the patterns, we describe three intersecting forces that shaped navigation across all conditions and countries: collective decision-making within extended family and peer networks, economic constraints and expectations of cure.

### Forces shaping navigation across all conditions

#### Collective decision-making and extended therapeutic networks

The first was the collective nature of illness management. Therapeutic decisions were rarely made individually: families financed care, directed treatment choices and sometimes over-rode patients’ own assessments. One patient in Burkina Faso described being steered away from hospital care despite his preference to continue treatment:

At the beginning of my diabetes, my sisters, my wife… said it was better to go and see a clairvoyant. It’s someone else who blamed me for leaving the hospital, because I’m going to waste my money. (patient, male, Burkina Faso)

This account shows a decision distributed across several actors who did not speak with one voice: the patient’s own preference to continue at the hospital, his sisters and wife steering him towards a clairvoyant, and a further relative criticising him for leaving hospital care as a waste of money. Collective decision-making was therefore not a single, settled position but an ongoing negotiation of difference within the family.

Digital platforms also emerged as influential spaces for advice. In Ghana, social media posts attributed to ‘Doctors’ sometimes promoted potentially harmful practices:

A doctor (on tik tok) also taught that, anyone with BP that shoots up to 200, buy Sprite, just the Sprite… (patient, female, Ghana)

In Niger, patient WhatsApp groups showed how group composition mattered. Smaller, medically moderated groups with clear rules for sharing information operated differently from larger open groups where patients, relatives, health workers and traditional healers all posted. As traditional healers became more visible in one open group, biomedical providers voiced concerns and then contributed less, while patients continued to engage with and act on messages they considered useful.

#### Economic constraints as the structural determinant of agency

The second force was financial. Economic barriers shaped not only whether patients could sustain biomedical care but also what form their agency could take when they could not. Across all three settings, participants described struggling with the direct costs of consultations and medicines, needing to travel to obtain drugs that were out of stock locally and losing income through repeated visits; some described selling possessions to fund biomedical investigations, only to move to traditional treatment when these costs became unsustainable.

When I have money, I go to the hospital; otherwise, I use plants. (patient, female, Niger)

These accounts depict cost as the backdrop against which other choices were made: patients did not leave biomedical care because they preferred traditional medicine in principle, but because escalating prices and access barriers left them with few viable options. Economic constraints did not remove patients’ capacity to act; they channelled agency into strategies such as switching sectors, alternating care over time or using herbs when hospital care was unaffordable.

#### Expectations of cure

The most consistent force was the mismatch between what biomedical chronic care offers and what patients sought. Patients across all three countries demonstrated biomedical literacy: they trusted diagnostic facilities, understood blood pressure and blood sugar readings and recognised the clinical authority of biomedical providers for establishing a diagnosis:

Traditionally, diabetes and hypertension cannot be detected. It’s when you go to the hospital to have your blood sample taken that the doctors will confirm… (patient, female, Burkina Faso)

Yet this trust did not translate into sustained engagement with biomedicine. Patients understood that biomedical management of hypertension and diabetes typically meant indefinite medication, not resolution, and this understanding drove consistent movement towards alternative providers who offered promises of an outright cure:

Both are helpful, but which one will make me stop taking the drugs altogether? The drugs are small, but taking them is difficult. It is bitter. So which one will cure it? (patient, male, Ghana)

Traditional healers across all three countries reinforced this expectation directly, positioning themselves as offering resolution where biomedicine offered only control—a claim patients encountered consistently, and that increased the credibility of the folk sector as an alternative destination. Movement away from biomedicine was therefore not a rejection of its diagnostic value; it was a rational response to its therapeutic limits. The prospect of cure was the most prominent, but not the only, driver: patients also turned to other sectors for the day-to-day management of specific symptoms and for dimensions of healing—social, spiritual and relational—that biomedical care did not address (a point developed in Pattern 2). Movement was thus a response to the perceived limits of biomedical management in several respects.

### Three patterns of therapeutic navigation

This section presents the three patterns of therapeutic navigation identified in the study. First, patients sequentially switched between sectors—mostly for cost reasons—as biomedical care became unaffordable. Second, patients used traditional and biomedical care concurrently, maintaining biomedical treatment while running self-managed traditional regimens. Third, for mental health, a spiritual-first trajectory dominated, in which explanations of spiritual causation and family decision-making shaped care-seeking regardless of the policy context. In each pattern, we examine the reasoning of those involved and how particular structural conditions shaped their navigation.

#### Pattern 1: sequential, cost-conditioned agency

When biomedical costs became untenable, patients did not abandon care; they sought care within the traditional sector. The most frequently described trajectory for hypertension and diabetes began with biomedical diagnosis and initial engagement, then shifted to traditional providers when the cumulative cost of consultations, laboratory monitoring and medicines exceeded what patients could sustain. Patients carried their biomedical diagnosis into the folk sector, using it as a foundation for seeking traditional treatment, and returned to biomedical care when resources allowed. What health systems recorded as discontinuation was a cost-driven movement between sectors. This pattern was most pronounced in Niger and Burkina Faso, where insurance coverage is minimal and out-of-pocket costs are highest, but was documented across all three countries.

When some people leave the medical for the traditional, it’s often not because they want to, it’s because they can’t afford it, but if you do the traditional treatment and God makes it easier, you’ll regain your health. (patient, male, Burkina Faso)

#### Pattern 2: concurrent use as pragmatic agency

A second pattern involved patients who maintained biomedical treatment while simultaneously adopting self-managed traditional regimens, thereby remaining in both systems rather than moving completely away from either. These patients assigned distinct functions to each sector: biomedicine was used for diagnosis, monitoring and symptom control, while traditional medicine was used to seek a cure and dimensions of healing that biomedicine did not address. They managed the interface between the two systems entirely without clinical oversight, for example, by carefully timing medicine dosing schedules to reduce perceived interaction risks:

If I take the medication for hypertension in the morning and the diabetes one in the morning, getting to the afternoon time, I will take the local one (herbs)… in the evening, I will still take the hospital one. (patient, male, Ghana)

This pattern operated across a spectrum determined by economic resources. For patients with limited means, concurrent use was a pragmatic strategy that combined the best available treatments from both sectors. When biomedical medications became unaffordable, traditional therapies filled the therapeutic gap without abandoning the biomedical pathway entirely. For patients with greater resources, concurrent use was motivated not by financial necessity but by conviction—the pursuit of a complete cure within the TCIM sectors that biomedical management could not promise. For these patients, spiritual care was valued independently from clinical outcomes and aligned with family and cultural community beliefs and preferences that biomedical encounters neither acknowledged nor engaged with.

A patient in Burkina Faso who incurred payments in both systems simultaneously captured both ends of this spectrum in a single reflection. Here, the patient describes paying simultaneously into both sectors—for biomedical consultations and medicines on one side and for traditional preparations (‘products’, the herbal remedies he purchases) on the other—and reflects on the cost this entails and on who could and could not afford it:

I can put 30,000 francs into products, and I know where I’m getting the money from, but can another citizen with a family of 05 people like that pay the 30,000 francs like me?… Both have benefits because I follow each treatment, and I’m gradually regaining my health. (patient, male, Burkina Faso)

Together, these accounts illustrate that concurrent use carries different meanings and is enabled by different conditions. Patients deliberately implemented parallel regimens with functions assigned to each sector; what differed was not the strategy itself but the degree of choice available within each sector.

#### Pattern 3: spiritual-first trajectories in mental health

Mental health conditions tended to generate a fundamentally different trajectory, with families and patients exercising agency within spiritual EMs from the outset. Patients attributed mental illness to jinn, curses, the evil eye and malevolent spiritual forces consistently across all three countries, and this framework directed families to traditional and spiritual healers before any question of biomedical services arose:

For me too, hospital treatment of diabetes and blood pressure is good and better, but for mental disorders, the ideal is traditional because it’s a delicate disease, so it’s better there. (patient, male, Burkina Faso)

Biomedical psychiatric services were often sought only after spiritual treatment had failed to bring sufficient improvement, or when deterioration forced crisis contact with biomedical services. This trajectory was consistent across all three countries, despite their different TCIM policies and mental health service configurations. What most strongly shaped where families turned first was their understanding of the condition’s cause and which type of treatment could address it, rather than the range of formally available services. This has direct implications for the design of mental health pathways, which need to engage with these explanatory frameworks rather than assuming that changing service availability alone will alter where help is sought first.

### Concealment as a relational outcome of clinical environments

Across all three patterns and countries, patients often concealed their pluralistic practices from biomedical providers. Patients’ accounts of concealment were corroborated by biomedical providers and by traditional and spiritual healers, who independently described the same behaviour and its triggers, allowing the two perspectives to be read against one another. Two distinct mechanisms created contexts for concealment across countries and conditions.

The first mechanism was provider disapproval, whether anticipated from reputation or experienced directly in previous encounters. When biomedical providers expressed strong opposition to traditional medicine on safety or professional grounds, patients concluded that disclosure would invite criticism rather than engagement and therefore remained silent. A physician in Burkina Faso described the consequence:

Often it’s because the traditional one didn’t work; they’re coming to the hospital right now for the last resort. They’ll never tell. (physician, female, Burkina Faso)

Where providers did voice objections, these were more often technical than principled.

We don’t know the composition of these drugs… It’s only in relation to dosage that my problem lies, and the side effects too. But as an African, I have no problem with traditional medicine. (physician, female, Burkina Faso)

Patients who had previously been criticised for using traditional medicine learnt not to disclose again. This was documented across all three countries, and within Ghana’s formally integrated system, specifically, where patients using licensed herbal medicine units reported being reprimanded by biomedical providers in the same facility:

We’ve had times where a client we were seeing had to go to the orthodox, and they were reprimanded for seeing us… So sometimes they are even afraid to actually mention that they were on herbal medicine. (medical herbalist, female, Ghana)

A positive finding was that in situations where providers created a safe space, patients were open to disclose their true trajectory:

It was after I’d asked the question properly, after I’d had a quiet chat with him, that he actually told me… He didn’t feel attacked. Because I told him the facts, and then I told him to draw his own conclusion. (physician, female, Burkina Faso)

The second mechanism was provider omission. Not all concealment was driven by fear; instead, in some encounters, the provider never asked, and the patient interpreted that silence as an absence of clinical interest:

I went to the hospital for my check-up, and the test showed that my blood pressure had dropped… Since he didn’t ask me, I didn’t tell him that, in addition to the tablets, I was taking the traditional medicine. He told me to continue with the product he prescribed, and that’s what I’m doing. (patient, male, Burkina Faso)

Some patients did not conceal out of fear but out of a realisation that their biomedical providers had neither the interest nor the capacity to engage with what they were doing:

I have not told them I take herbal medicine… It is because it is not needful to them. I am the one who needs it, or don’t you understand? (patient, male, Ghana)

Consultations thus produced apparent concordance while the patient’s actual therapeutic strategy remained entirely unexamined.

Taken together, these mechanisms demonstrate that concealment is not merely a patient behaviour problem. It is also a systems problem, produced and sustained by clinical environments that are not designed to encourage disclosure and to engage with patients’ non-conventional pathways. The findings from Ghana in particular decisively sharpen this point. Formal TCIM integration created additional service options without altering the underlying conditions driving concealment. Provider attitudes, clinical omissions, fear of reprimand and patients’ calculations about what disclosure achieves are not altered by regulatory frameworks or co-located services.

## Discussion

Our study examined how and why patients and families adapt care strategies across biomedical, traditional and spiritual sectors for hypertension, diabetes and mental health in Burkina Faso, Ghana and Niger, and what those strategies reveal about patient agency and the conditions needed for safer, more effective chronic care. The three patterns of therapeutic navigation identified show that patients and families exercise considerable agency, assigning functions to different sectors, managing costs and timing, and making decisions that current NCD care frameworks neither recognise nor support.

### What patients are doing, and why

Existing work on medical pluralism in Africa typically highlights poverty, cultural beliefs, dissatisfaction with biomedical services or weak systems as primary drivers of TCIM use. The three patterns identified here indicate that these factors operate together rather than in isolation. Patients with hypertension and diabetes who could not sustain biomedical costs often relocated care to traditional providers while carrying their diagnoses and prior treatment experience, consistent with adherence studies that identify cost and medicine availability as key reasons for discontinuation.^46^ Better-off patients sometimes maintained concurrent use, relying on biomedical care for diagnosis and monitoring and on traditional therapies for cure, protection or symptom relief. For mental health, families commonly turned first to spiritual and traditional healers regardless of costs or service availability, as reported in other West African work.^47^

This extends sector-based models of healthcare by showing how structural conditions shape the form of choice across sectors. It aligns with Emirbayer and Mische’s view of agency as iterative, projective and practical–evaluative.^16^ In our data, patients iteratively used experience to recalibrate sector choice, pursued cure or spiritual protection when management was projected as inadequate, and actively timed and combined therapies based on perceived risks and benefits. Economic resources influenced how this agency could be exercised, not whether it existed.

Furthermore, the choice of care across sectors is shaped by the inability of local health systems to become responsive to people’s medical and non-medical expectations,^48^ such as the expectations of cure found in this study. A deeper understanding of the underlying mechanisms of health systems responsiveness, such as trust in local health systems and services,^49^ is critical to ensuring truly responsive health systems that recognise and address people’s needs.

The tension between cure and lifelong management was central to navigation and cannot be explained by ‘poor health literacy’ alone. Patients understood and valued biomedical diagnosis, yet many continued to seek a cure. In Hausa, commonly spoken in Niger and northern Nigeria, the common rendering of ‘chronic’ evokes a condition that is difficult to treat rather than something managed indefinitely, echoing ethnographic work showing that local naming of diabetes and hypertension is grounded in symptom experience and expectations of cure.^50 51^ Transposing the chronic care model into contexts where these assumptions differ raises questions that education alone cannot resolve. Traditional healers’ credibility rested partly on their willingness to offer the prospect of an ending; when this logic remains outside clinical awareness, it increases the risk of unmonitored combinations and delayed escalation of care.

### Collective decision-making and extended networks

Families often financed NCD care, directed treatment choices and sometimes over-ruled patients’ assessments, consistent with wider evidence that kin play a central role in health decision-making in African households.^52 53^ Patients who could self-fund biomedical visits sometimes used this to maintain their preferred trajectories; others were more dependent on relatives to attend appointments and obtain medicines.

Digital platforms extend these family and peer dynamics into information spaces that largely sit outside clinical oversight. Social media operate in the popular sector, circulating health content that patients may treat as authoritative, but that is unevenly regulated. Evidence suggests that a substantial share of hypertension-related online content promotes alternative remedies and often lacks scientific grounding, mirroring our Ghana findings that some patients act on advice attributed to unverified ‘doctors’ in ways that may be unsafe.^54^ Accounts from the medically moderated and open WhatsApp groups in Niger illustrate how the composition and norms of digital spaces shape whose voices are heard: as traditional healers became more prominent in some open groups, biomedical providers tended to withdraw while patients continued to consume and act on available information. The broader implication is that digital networks can amplify pluralistic care-seeking while making it harder for health professionals to remain engaged, raising questions about how health systems can support patient safety in information environments they do not control.

### Concealment, safety and systems failure

Concealment is where the safety implications of these dynamics become most acute. Patients across all three settings combined biomedical and traditional therapies, adjusting timing and dosage without clinical guidance on interactions or contraindications, echoing broader concerns about herb–drug interactions in NCD care.^55^ The point is not to discourage the use of traditional medicine, which will continue, but to make concurrent use sufficiently visible to enable discussion of risks and coordination.

Two mechanisms driving concealment—provider disapproval and provider omission—reflect features of clinical environments rather than simple patient reluctance. Fear of reprimand and possible retribution is a critical determinant of people’s (non) engagement with health systems,^56 57^ and effective feedback mechanisms are essential to establishing transparent, reliable and responsive systems in which patient voices can be heard.^58^ Our findings illustrate the limits of current integration approaches in places like Ghana. Despite herbal units, regulatory structures and co-located services, patients still described reprimand for consulting traditional healers. Concealment dynamics there resembled those in Burkina Faso and Niger, suggesting that integration efforts narrowly focused on regulation and co-location address a supply-side problem (formal access to traditional services) but leave the demand-side problem (the safety and usefulness of disclosing actual practices) largely untouched.

### Implications for policy: recognition, tolerance and dialogue

Our findings point to three immediate priorities for policy makers and service planners: recognition, tolerance and dialogue. Recognition means acknowledging that patients already coordinate care across sectors and that health systems rely on this work without supporting it. Tolerance involves treating pluralistic strategies as understandable responses to constraints and expectations, even when they do not align with biomedical norms. This does not imply that all traditional practices are safe, but it does require beginning from an assumption of rationality rather than deviance.

Dialogue calls for clinical encounters in which asking about other forms of care is routine, non-judgmental and explicitly linked to concerns about safety and effectiveness rather than blame. Evidence suggests that it is the content and relational quality of consultations—space to explore psychosocial concerns, negotiate treatment plans and build trust—rather than duration alone, that improves communication and creates conditions more conducive to honest disclosure.^59 60^ In practical terms, provider training should be coupled with consultation protocols that include standard, non-judgmental prompts about traditional and spiritual care framed around safety and coordination. Health information systems could record whether pluralistic practice was discussed, not just whether patients attended appointments.

Furthermore, system indicators should distinguish between abandonment of care and continued care elsewhere, so that loss to follow-up is not conflated with therapeutic navigation. Given families’ central roles in financing and decision-making, involving key relatives in discussions about treatment options and safety is an obvious, low-cost intervention. For mental health, structured engagement with spiritual and traditional healers around referral and risk-sharing is particularly important, given their role as first-line providers in many cases.

## Limitations

Recruitment largely through health facilities means the sample likely over-represents patients who have engaged with biomedical care and under-represents those whose trajectories lie entirely within traditional and spiritual sectors; this was partly mitigated by including traditional and spiritual healers and caregivers, whose accounts provided perspectives from outside biomedical settings. The number and types of participants varied across countries, limiting direct comparisons between specific conditions and provider groups. Interviewing patients, providers, healers and caregivers within each country helped maintain coherent findings where cross-country comparisons were not possible. Patients may also have under-reported their use of traditional and spiritual care to researchers, just as the findings show they do with clinicians. Where possible, patient accounts were cross-checked against those of providers and healers to assess where these aligned and where they differed. Finally, the analysis is cross-sectional; although data collection occurred at a single point in time, participants narrated care trajectories retrospectively, and the iterative abductive approach, involving multiple rounds of coding and conceptual revision across the full team, supported sufficient analytical depth despite this constraint.

## Conclusions

This study shows that pluralistic NCD care-seeking should not be interpreted as failed adherence, but as a widespread and deliberate form of patient and family agency operating under structural constraints. Across settings, patients do not abandon care when they move beyond biomedical services; rather, they navigate between biomedical, traditional and spiritual sectors; carry diagnoses across systems; assign distinct therapeutic roles to each and manage combined treatments that health systems often neither recognise nor support. These patterns reveal a broader mismatch between linear models of chronic care and the realities of healthcare pluralism common in many health systems globally, shaped by collective decision-making, economic constraints and expectations of cure.

A key lesson for the wider literature is that regulatory integration and service co-location alone are insufficient to address the risks associated with pluralistic care. Even where traditional medicine is formally incorporated into public facilities, concealment persists when clinical encounters, financing arrangements and professional norms fail to make disclosure safe or meaningful. Concealment thus emerges not as an individual failing, but as a predictable outcome of systems that privilege biomedical singularity while leaving patients to manage chronic care journeys alone.

Health systems need to distinguish disengagement from care from continued care elsewhere, move beyond adherence indicators that classify navigation as failure, reduce cost-driven switching through improved financing and create consultation spaces that invite non-judgmental discussion of pluralistic practice. More broadly, we call for health systems research to treat pluralism as a constitutive feature of care rather than a residual problem, recognising patients as active agents managing illness across multiple therapeutic worlds.

## Supplementary material

10.1136/bmjopen-2026-123607 online supplemental file 1

10.1136/bmjopen-2026-123607 online supplemental file 2

## Data Availability

Data are available upon reasonable request.
