# Supplementary material for: Beyond non-adherence: qualitative insights into how patients strategically navigate pluralistic chronic care in West Africa
Source: BMJ Open. 2026 Oct 15;16(10):e123607. doi: 10.1136/bmjopen-2026-123607 (PMC13691745; doi:10.1136/bmjopen-2026-123607)
Supplement: Supplementary file 1 [file bmjopen-2026-123607.reviewer_comments.pdf]

PEER REVIEW HISTORY

BMJ Open publishes all reviews undertaken for accepted manuscripts. Reviewers are asked to complete a checklist review form and are provided with free text boxes to elaborate on their assessment. These free text comments are reproduced below.

ARTICLE DETAILS

Title (Provisional)

Beyond non-adherence: qualitative insights into how patients strategically navigate pluralistic chronic care in West Africa

Authors

Abdul-Samed, Abdul-Basit; Mamad, Ousseini Teoma; Kabore, Anicet Tounwendsida; Amarteyfio, Kezia Naa Amerley Akosua; Yamba, Salamatou Abdoul Karim; Diarra, Aissa; Yaogo, Maurice; Owusu-Ansah, Angela; McKee, Martin; Mirzoev, Tolib; Agyepong, Irene Akua; Yevoo, Lucy; Spicer, Neil; Koduah, Augustina; Balabanova, Dina

---

VERSION 1 - REVIEW

**Reviewer**	**1**
**Name**	**Reich, Amanda**
**Affiliation**	**Brigham and Women's Hospital**
**Date**	**29-Jun-2026**
**COI**	**None**

Thank you for the opportunity to review this manuscript. It describes an important contribution to the literature; a qualitative case study examining non-communicable disease pathways across biomedical, traditional, and spiritual sectors in three African countries.

Methods: conceptual frameworks. Please clarify the use of the Kleiman's health system model, theory of agency, and therapeutic itineraries framework. How were all 3 frameworks actually integrated in the analysis? Were they used to develop the instruments and define domains, or to structure the analysis of data? You discuss some of this in the analysis section, but its difficult to interpret how all three frameworks guided the analysis.

It would be helpful to have a description of the interview guide, its development, and more detail on key domains included and described. Did guides differ between patients, caregivers, providers, healers, and policymakers?

Why were the three conditions described (hypertension, diabetes, and mental health) selected as the NCDs to include?

Results

The themes focus on important patient perspectives, but the role of other participants (providers, healers) doesn't really show up until concealment is described. It is unclear where policymakers influenced the analysis. Were the other themes also analyzed across strata? For example, fears of reprimand or concealment was described by patients, but did any providers clearly confirm or deny this behavior from their own perspectives? It is a strength of the study that the data allow comparison across cases as well as roles, but it seems like there is a missed opportunity to further develop these different perspectives and potentially triangulate some of the data.

**Reviewer**	**2**
**Name**	**Rapley, Tim**
**Affiliation**	**University of Northumbria at Newcastle, Social Work, Education and Community Wellbeing**
**Date**	**31-Jul-2026**
**COI**	**None**

Really, really, lovely and well argued, conceptually informed (re)specification of 'non-adherence', how a such pluralistic health seeking practices are embedded in collective, deliberative, adaptive practice.

Strong (conceptual) framing of results – moving from contextual (expectations of care, economics, and distributed decisional infrastructures) to the three trajectories, with a strong focus on (re)thinking conditions of possibility around practical, structural, professional conditions of concealment to enable different conversations (recognition, tolerance, dialogue).

I'm only going to focus on outlining some small points (pretty rare for me, I'm normally much more annoying!)

Very (very) minor points

In the 'expectations of cure' – is this the only route to seeking alternates, as in TCIM as not only about 'cure' but about, say, 'managing symptoms' in alternate ways? It seems too totalising to see therapeutic limits as only tied to lack of cure (and lack of hope), as opposed to say more mundane reasons, so lack of managing some specific aspect on a day-to-day way. You raise something like this in 'Pattern 2' – when you refer to 'dimensions of healing that biomedicine did not address' – which feels much more coherent addition/caveat to this section.

In the 'economic constraints' section - do we need three quotes when one will do – this is strong enough, very strongly echoes prior work, etc. Also, as the final context section

outlines, this is rarely solo decision – rarely just 'patients' capacity to act', but rather, rather networks embedded in. I would then be tempted to start with 'collective decision-making' – as this then shapes/enmeshed with how the other two can be read.

In 'collective' the first quote needs unpacking – we see the intro render as his preference, then sister and wife, then a third (collective) actor appears 'someone else' – and that might need explaining and/or delete it (also, what is nice here is how this collective may not be unitary, constantly, managing difference and emergence over time).

In 'Pattern 2' – I'm not sure the second quote does the work without some more context/explanation – so what are the elements of the two systems – is 'products' a reference to what he gains from both.

In 'concealment', in second sentence, I'm not sure what you describe are 'two distinct forms' but rather, two distinct mechanisms that can create contexts of/for concealment (and you use 'mechanism' later).

VERSION 1 - AUTHOR RESPONSE

Reviewer 1 (Dr Amanda Reich)

Comment	Author response
Thank you for the opportunity to review this manuscript. It describes an important contribution to the literature; a qualitative case study examining non-communicable disease pathways across biomedical, traditional, and spiritual sectors in three African countries.	We thank the reviewer for these encouraging comments and for a set of questions that have materially improved the methods reporting.
Methods: conceptual frameworks. Please clarify the use of Kleinman's health system model, theory of agency, and therapeutic itineraries framework. How were all 3 frameworks actually integrated in the analysis? Were they used to develop the instruments and define domains, or to structure the analysis of data? You discuss some of this in the analysis section, but it's difficult to interpret how all three frameworks guided the analysis.	To answer the reviewer's question directly, the three frameworks were used to structure the analysis rather than to design the data-collection instruments (the topic guides were developed by the country teams around shared domains and refined through community engagement; see our response to the next comment). Within the analysis, each framework played a distinct but complementary role: Kleinman's healthcare-sectors model provided the initial deductive coding frame, distinguishing the popular, folk and professional sectors and separating biomedical disease from lived illness through explanatory models; the therapeutic-itineraries framework structured the longitudinal reading of the data, allowing us to trace how care-seeking unfolded and was adjusted over time and to identify the recurring patterns of navigation; and Emirbayer and Mische's theory of agency provided the interpretive lens for explaining why patients and families moved between sectors as they did.

	We have revised the Analysis section to make these distinct roles explicit.
It would be helpful to have a description of the interview guide, its development, and more detail on key domains included and described. Did guides differ between patients, caregivers, providers, healers, and policymakers?	Yes - role-specific guides were used, and we have expanded the description accordingly. Rather than a single harmonised instrument, each country team developed and adapted its own semi-structured topic guides, in the relevant languages, around a common set of domains agreed across the consortium and informed by community engagement. Within each country, distinct versions were used for patients and caregivers, biomedical providers, traditional and spiritual healers, and policymakers.<br><br>Common domains covered illness narratives and beliefs about causation; care-seeking trajectories and movement between biomedical, traditional and spiritual sectors; family and social influences on decision-making; economic barriers; and perceptions of the efficacy and safety of each type of care. Developing and adapting guides locally preserved linguistic, cultural and role-appropriate framing across very different settings, while the shared domains and common analytical frameworks maintained comparability at the analysis stage. These points are now set out in the Sampling and data collection section.
Why were the three conditions described (hypertension, diabetes, and mental health) selected as the NCDs to include?	These are the three conditions included in the study's funding proposal, which sets out the rationale for choosing them in detail. In brief, all three are highly prevalent, frequently co-exist, and share modifiable risk factors as well as overlapping challenges in access, continuity of care and long-term management - and this is also true in the three study countries. Hypertension affects about one in four adults in Ghana, with similarly high and rising prevalence across Burkina Faso and Niger, especially in urbanising regions. Diabetes is a major contributor to premature mortality and catastrophic household expenditure in all three countries. Both conditions are strongly linked to common mental health disorders, which, when we were developing the proposal, policymakers emphasised to us as integral to chronic disease management; those in all three countries highlighted the importance of addressing stress, anxiety and depression because they directly influence treatment adherence, self-management and patient–provider relationships. Together, these NCDs represent a cluster of conditions that are (1) highly burdensome, (2) amenable to primary-care strengthening and task-shifting, (3) central to national NCD strategies, and (4) responsive to community-based support and improved access to medicines.

	Adding all of this detail would lengthen the paper appreciably, so we have added the following concise text to the manuscript: "These three conditions were selected in consultation with policymakers in the countries concerned on the basis of their long-term health and economic burden, their frequent co-existence, and the potential to improve their management." However, if this is insufficient, we would be glad to expand the text.
Results: The themes focus on important patient perspectives, but the role of other participants (providers, healers) doesn't really show up until concealment is described. It is unclear where policymakers influenced the analysis. Were the other themes also analyzed across strata? For example, fears of reprimand or concealment was described by patients, but did any providers clearly confirm or deny this behavior from their own perspectives? It is a strength of the study that the data allow comparison across cases as well as roles, but it seems like there is a missed opportunity to further develop these different perspectives and potentially triangulate some of the data.	We have made the cross-role analysis explicit. Patient and family accounts lead the Results because the paper's analytical claim concerns patient and family agency; however, all themes — not only concealment — were coded and analysed across participant strata (patients, caregivers, biomedical providers, healers and policymakers) and across the three countries, and provider, healer and policymaker accounts were used throughout to corroborate, qualify or complicate patient accounts. We have added a sentence to the Analysis section stating this.<br><br>Provider and healer accounts do in fact confirm the concealment patients described — for example, the physician in Burkina Faso ("…They'll never tell.") and the medical herbalist in Ghana who described patients being reprimanded in the same facility. We have signposted this corroboration at the start of the concealment section rather than leaving it implicit, and we now note where accounts converged and diverged.<br><br>Policymakers' accounts principally informed the study's contextual framing and the analysis of integration and of digital-information governance (for example, the Niger WhatsApp-group observations and the interpretation of formal integration in Ghana); we have made this contribution clearer in the Results and Discussion.

Reviewer 2 (Prof. Tim Rapley)

Comment	Author response
Really, really, lovely and well argued, conceptually informed (re)specification of 'non-adherence'… Strong (conceptual) framing of results… I'm only going to focus on outlining some small points (pretty rare for me, I'm normally much more annoying!)	We are grateful for these generous comments and have addressed each of the specific points below.
In the 'expectations of cure' – is this the only route to seeking alternates, as in TCIM as not only about 'cure' but about, say, 'managing symptoms' in alternate ways? It seems too totalising to see therapeutic limits as only tied to lack of cure (and lack of hope)… You raise	We agree this was too totalising and have added a caveat, drawing on the Pattern 2 framing the reviewer helpfully identifies. The 'Expectations of cure' section now notes that the prospect of cure was the most prominent but not the only driver, and that patients also turned to other sectors for the day-to-day management of specific

something like this in 'Pattern 2' – when you refer to 'dimensions of healing that biomedicine did not address' – which feels much more coherent addition/caveat to this section.	symptoms and for social, spiritual and relational dimensions of healing that biomedical care did not address.
In the 'economic constraints' section – do we need three quotes when one will do… Also, as the final context section outlines, this is rarely solo decision… I would then be tempted to start with 'collective decision-making' – as this then shapes/enmeshed with how the other two can be read.	Agreed on both counts. We have reduced the economic-constraints quotations from three to one, retaining the most economical illustration (and keeping the narrative point of the deleted quotation in prose), and we have reordered the three forces to begin with collective decision-making, so that it frames the reading of economic constraints and expectations of cure. The corresponding lists in the abstract, the Results preamble and the Conclusion have been updated to match the new order.
In 'collective' the first quote needs unpacking – we see the intro render as his preference, then sister and wife, then a third (collective) actor appears 'someone else' – and that might need explaining… (also, what is nice here is how this collective may not be unitary, constantly, managing difference and emergence over time).	We have added an unpacking sentence after the quotation that names the different actors and their competing positions - the patient's own wish to continue at the hospital, his sisters and wife urging him toward a clairvoyant, and a further relative warning him against leaving hospital care for fear of wasting money - and makes explicit that the therapeutic network was not a single, settled position but an ongoing negotiation of difference within the family.
In 'Pattern 2' – I'm not sure the second quote does the work without some more context/explanation – so what are the elements of the two systems – is 'products' a reference to what he gains from both.	We have added a lead-in that explains what the patient is paying for in each sector — biomedical consultations and medicines on one side, and traditional preparations ('products', the herbal remedies he purchases) on the other — and clarifies that he pays into both sectors simultaneously.
In 'concealment', in second sentence, I'm not sure what you describe are 'two distinct forms' but rather, two distinct mechanisms that can create contexts of/for concealment (and you use 'mechanism' later).	Agreed; this aligns the terminology with the rest of the section, which already refers to 'mechanisms'. We have changed "Two distinct forms of concealment operated…" to "Two distinct mechanisms created contexts for concealment…", and "The second form was provider omission" to "The second mechanism was provider omission."

VERSION 2 - REVIEW

Reviewer 2

Name Rapley, Tim

Affiliation University of Northumbria at Newcastle, Social Work, Education and Community Wellbeing

Date **24-Sep-2026**

COI

A great paper. I think you nicely responded to our questions/queries.
