# Supplementary material for: Beyond non-adherence: qualitative insights into how patients strategically navigate pluralistic chronic care in West Africa
Source: BMJ Open. 2026 Oct 15;16(10):e123607. doi: 10.1136/bmjopen-2026-123607 (PMC13691745; doi:10.1136/bmjopen-2026-123607)
Supplement: Supplementary file 2 [file bmjopen-16-10-s001.docx]

**Supplementary Table S2. Consolidated Criteria for Reporting Qualitative Studies (COREQ) — 32-Item Checklist**

Manuscript: Beyond non-adherence: qualitative insights into how patients strategically navigate pluralistic chronic care in West Africa (bmjopen-2026-123607)

Source: Tong A, Sainsbury P, Craig J. Consolidated criteria for reporting qualitative research (COREQ): a 32-item checklist for interviews and focus groups. Int J Qual Health Care. 2007;19(6):349–357.

*Note: Page numbers refer to the revised manuscript (clean copy). Items reported in this checklist rather than in the main text are marked accordingly. Please re-verify page numbers against the final paginated PDF before submission.*

| **No.** | **Item** | **Guide question / description** | **Reported location / response** | **Page(s)** |
| --- | --- | --- | --- | --- |
| **DOMAIN 1: RESEARCH TEAM AND REFLEXIVITY** | | | | |
| ***Personal Characteristics*** | | | | |
| 1 | **Interviewer / facilitator** | Which author/s conducted the interview or focus group? | Semi-structured interviews were conducted by the authors and country-based, trained research assistants under the supervision of country co-investigators.  Ghana (supervised by LLY, KAA): Abdul-Basit Abdul-Samed, Kezia Amerley Amarteyfio, Nhyira Adjei Banuah, Nana Efua Enyimayew Afun, Abena Boahema Edusei, Grace Emmanuelle Victoire Aye, Dominic Anaseba.  Burkina Faso (supervised by ATK and MY): Anicet Tounwendsida Kabore, Koudougou Vincent, Konkobo Mathias, Sana Lisata, ZAGRE Cynthia, Sanon Vincent Paul, Diabagate Aziz, Dah Noemie, Sarah Rachel, Zoungrana Pascal.  Niger (supervised by OTM and AD): Ousseini Teoma Mamad, Salamatou Yamba, Babba Gana Elhadji Moustapha CHETIMA, Saidou Oumarou Mahamane, Farouck Adamou Gaoh.  See: Acknowledgements; Methods – Sampling and data collection. | **pp. 7, 19** |
| 2 | **Credentials** | What were the researcher's credentials? e.g. PhD, MD | ABAS (MD, MSc); OTM (MSc); ATK (PhD, MSc); KAA (MD); SY (MSc); AD (PhD); MY (PhD); AOA (PhD); MMK (DSc, MD); TM (PhD); IAA (DrPH, MD); LLY (PhD); NS (PhD); AK (PhD); DB (PhD).  Field researchers held Bachelor's or Master's-level qualifications in Medicine, public health, social sciences, or related disciplines. | **pp. 20–21; this checklist** |
| 3 | **Occupation** | What was their occupation at the time of the study? | ABAS – Public health researcher, Ghana College of Physicians and Surgeons (GCPS), Accra, Ghana.  OTM – Researcher, LASDEL, Niamey, Niger.  ATK – Researcher, UCAO-UUB, Bobo-Dioulasso, Burkina Faso.  KAA – Public health researcher, GCPS, Accra, Ghana.  SY – Researcher, LASDEL, Niamey, Niger.  AD – Doctor of Medicine, LASDEL, Niamey, Niger.  MY – Senior Researcher, UCAO-UUB, Bobo-Dioulasso, Burkina Faso.  AOA – Professor of Behavioural and Social Science, Ashesi University, Berekuso, Ghana.  MMK – Professor of European Public Health, LSHTM, London, UK.  TM – Professor of Global Health Policy, LSHTM, London, UK.  IAA – Professor of Public Health, GCPS; Senior Researcher, Dodowa Health Research Centre, Ghana Health Service, Ghana.  LLY – Senior Researcher, Ghana College of Physicians and Surgeons.  NS – Associate Professor of Global Health Policy, LSHTM, London, UK.  AK – Associate Professor, School of Pharmacy, University of Ghana, Legon, Ghana.  DB – Professor of Health Systems and Policy, LSHTM, London, UK.  See: Authors' Affiliations. | **pp. 20–21; this checklist** |
| 4 | **Gender** | Was the researcher male or female? | ABAS – Male. OTM – Male. ATK – Male. KAA – Female. SY – Female. AD – Female. MY – Male. AOA – Female. MMK – Male. TM – Male. IAA – Female. LLY – Female. NS – Male. AK – Female. DB – Female. | **this checklist** |
| 5 | **Experience and training** | What experience or training did the researcher have? | All country-based research assistants received standardised pre-fieldwork training covering qualitative interviewing techniques, informed consent procedures, confidentiality, and data management protocols. Training was delivered by country co-investigators.  Country co-investigators (ABAS, OTM, ATK) have extensive experience conducting qualitative research in health systems contexts in West Africa.  Senior authors have multi-decade experience leading multi-country qualitative and health systems research globally and in sub-Saharan Africa. | **p. 7; this checklist** |
| ***Relationship with participants*** | | | | |
| 6 | **Relationship established** | Was a relationship established prior to study commencement? | No prior relationship existed between interviewers and participants. Participants were introduced to the research team through facility managers, district health officers, or community gatekeepers, as appropriate to each setting. |  |
| 7 | **Participant knowledge of the interviewer** | What did the participants know about the researcher? e.g. personal goals, reasons for doing the research | At the outset of each interview, participants were informed about: the purpose and objectives of the study; the institutional affiliation of the research team; the voluntary nature of participation; their right to withdraw at any time without consequence; and how their data would be used and stored. This was delivered through the written and verbal informed consent process, conducted in the participant's preferred language (English, French, or local language).  See: Methods – Ethics. | **p. 8** |
| 8 | **Interviewer characteristics** | What characteristics were reported about the interviewer/facilitator? e.g. bias, assumptions, reasons and interests in the research topic | Researchers were selected for their language proficiency (English, French, Twi, Hausa, Zarma, Mooré, Dioula) and cultural familiarity with the study communities to support rapport and communication.  Pre-fieldwork training addressed potential interviewer assumptions, the need for non-directive probing, and the importance of eliciting participant perspectives without imposing analytical categories.  No prior relationship existed between interviewers and participants. | **p. 7;** |
| **DOMAIN 2: STUDY DESIGN** | | | | |
| ***Theoretical framework*** | | | | |
| 9 | **Methodological orientation and theory** | What methodological orientation was stated to underpin the study? e.g. grounded theory, discourse analysis, ethnography, phenomenology, content analysis | Multiple qualitative case study design.  Three interconnected theoretical frameworks guided analysis of the study:  1. Kleinman's healthcare systems model – structuring analysis across popular, folk, and professional sectors and drawing on the concept of explanatory models.  2. Emirbayer and Mische's theory of agency – conceptualising agency as temporally embedded, collectively shaped, and adaptive rather than purely individual and rational.  3. The therapeutic itineraries framework – tracing patient pathways across sectors over time.  Data were analysed using abductive thematic analysis. | **p. 5** |
| ***Participant selection*** | | | | |
| 10 | **Sampling** | How were participants selected? e.g. purposive, convenience, consecutive, snowball | Purposive sampling was used across all three countries to ensure representation of: (a) different disease types (hypertension, diabetes, common mental health conditions); (b) different participant types (patients/caregivers, biomedical providers including medical herbalists, traditional and spiritual healers, policymakers); and (c) variation across urban and rural settings within each country. | **p. 6** |
| 11 | **Method of approach** | How were participants approached? e.g. face-to-face, telephone, mail, email | Face-to-face approach. Participants were identified and recruited through health facilities (outpatient departments, NCD clinics), community settings, and via gatekeepers, including facility managers, district health officers, and community leaders, as appropriate to participant type and setting. | **p. 6** |
| 12 | **Sample size** | How many participants were in the study? | 84 participants in total, across three countries:  • People with hypertension, diabetes, or common mental health conditions: n=29  • Biomedical providers (including medical herbalists): n=29  • Traditional and spiritual healers: n=13  • Family members/caregivers: n=3  • National-level policymakers: n=10 | **pp. 2, 6** |
| 13 | **Non-participation** | How many people refused to participate or dropped out? Reasons? | No dropout was encountered across the three country fieldwork sites. | this checklist |
| ***Setting*** | | | | |
| 14 | **Setting of data collection** | Where was the data collected? e.g. home, clinic, workplace | Data were collected across 11 purposively selected municipalities and districts:  Ghana: Ga South Municipality and Ledzokuku Municipality (Greater Accra Region).  Burkina Faso: Dô and Dandé (Hauts-Bassins Region); Koudougou and Réo (Centre-Ouest Region).  Niger: Communal Arrondissement 5 (Niamey); Tahoua Commune, Madaoua, 17 Portes, and Mayahi.  Settings included public health facilities (outpatient/NCD clinics), herbal medicine units, community settings, and participants' homes. | **pp. 4, 6** |
| 15 | **Presence of non-participants** | Was anyone else present besides the participants and researchers? | Interviews were conducted on a one-to-one basis between the interviewer and participant. Where culturally appropriate and with the participant's explicit consent, a family caregiver was permitted to be present. No other non-participants were present. | **this checklist** |
| 16 | **Description of sample** | What are the important characteristics of the sample? e.g. demographic data, date | The sample comprised five participant types across three countries (Ghana, Burkina Faso, Niger), covering three disease areas (hypertension, diabetes, common mental health conditions). Participants included both male and female (by sex) respondents across a range of ages and socioeconomic circumstances.  Data collection was conducted in 2023 under Phase 1 of the STOP-NCD consortium.  See: Methods – Table 1. | **p. 6** |
| ***Data collection*** | | | | |
| 17 | **Interview guide** | Were questions, prompts, guides provided by the authors? Was it pilot tested? | Semi-structured interview topic guides were developed by the multi-country research team, led by ABAS, OTM, and ATK, with inputs from country co-investigators.  Guides covered: illness narratives and explanatory models; care-seeking trajectories across biomedical, traditional, and spiritual sectors; family and social influences on care decisions; economic factors; and perceptions of efficacy and safety.  Separate guides were developed for each participant type. Guides were produced in English, translated into French and five local languages (Twi, Hausa, Zarma, Mooré, Dioula), and pilot tested with small groups in each country before fieldwork commenced; findings from pilot testing informed revisions. | **p. 7** |
| 18 | **Repeat interviews** | Were repeat interviews carried out? If yes, how many? | Repeat interviews were not conducted. |  |
| 19 | **Audio/visual recording** | Did the research use audio or visual recording to collect the data? | Yes. All interviews were audio-recorded with participants' prior informed consent. Recordings were subsequently transcribed verbatim.  See: Methods – Sampling and data collection. | **p. 7** |
| 20 | **Field notes** | Were field notes made during and/or after the interview or focus group? | Yes. Research assistants recorded contextual and reflective field notes after each interview, capturing observations about the interview setting, any notable interactional dynamics, and initial analytical reflections. Field notes were shared with country co-investigators and contributed to team-based analytical discussions. | **this checklist** |
| 21 | **Duration** | What was the duration of the interviews or focus group? | Interviews ranged from approximately 30 to 90 minutes, depending on participant type and the depth of discussion.  See: Methods – Sampling and data collection. | **p. 7** |
| 22 | **Data saturation** | Was data saturation discussed? | Data saturation was applied: data collection continued until saturation. | **p. 8** |
| 23 | **Transcripts returned** | Were transcripts returned to participants for comment and/or correction? | No. Transcripts were not returned to participants. Given the three-country, multilingual, multi-site design, formal member-checking of transcripts was not feasible. Rigour was maintained through: team-based coding across country investigators; iterative cross-country analytical meetings; and review of interpretations by all co-authors. |  |
| **DOMAIN 3: ANALYSIS AND FINDINGS** | | | | |
| ***Data analysis*** | | | | |
| 24 | **Number of data coders** | How many data coders coded the data? | Primary coding was led by ABAS, with structured contributions from country co-investigators (OTM, ATK) coding transcripts from their respective country datasets.  All emerging analytical categories and interpretations were reviewed iteratively by the full authorship team through a series of cross-country meetings.  See: Methods – Analysis. | **pp. 7–8; this checklist** |
| 25 | **Description of the coding tree** | Did authors provide a description of the coding tree? | The coding structure was developed in two stages:  Stage 1 (deductive): Initial codes were applied using Kleinman's healthcare sector model (popular, folk, professional sectors), generating sector-level categories aligned with the conceptual framework.  Stage 2 (inductive/abductive): Codes were iteratively refined through close reading of the data, generating the three navigation patterns (sequential cost-driven switching; concurrent biomedical and traditional use; spiritual-first trajectories) and the cross-cutting finding on concealment. | **pp. 5, 7–8** |
| 26 | **Derivation of themes** | Were themes identified in advance or derived from the data? | Both. Initial codes were deductively structured using Kleinman's model. Themes were subsequently derived inductively through abductive iteration between the empirical data and the three theoretical frameworks (Kleinman, Emirbayer and Mische, therapeutic itineraries). The three navigation patterns and concealment emerged from the data through this iterative process rather than being imposed in advance.  See: Methods – Analysis. | **pp. 7–8** |
| 27 | **Software** | What software, if applicable, was used to manage the data? | NVivo 12 (QSR International) as well as Microsoft Excel was used for data organisation, management, and coding. | **p. 8** |
| 28 | **Participant checking** | Did participants provide feedback on the findings? | Given the multi-country, multilingual design and the analytical abstraction involved in moving from individual accounts to cross-country patterns, returning findings to individual participants was not feasible.  Findings will be disseminated through country research teams and Stop-NCD partner organisations, engaging a stakeholder network that includes patient and community representatives, in line with the consortium's patient and public involvement commitments. | **p. 8** |
| ***Reporting*** | | | | |
| 29 | **Quotations presented** | Were participant quotations presented to illustrate the themes/findings? Was each quotation identified? e.g. participant number | Yes. Direct participant quotations are presented throughout the Results section to illustrate each of the three navigation patterns and the cross-cutting concealment finding.  Each quotation is attributed by participant type, gender, and country (e.g. 'Patient, Male, Burkina Faso'; 'Medical Herbalist, Female, Ghana'; 'Physician, Male, Burkina Faso'), consistent with the study's ethical commitment to participant confidentiality while providing interpretive context. | **pp. 8–14** |
| 30 | **Data and findings consistent** | Was there consistency between the data presented and the findings? | Each of the three navigation patterns and the concealment finding is grounded in multiple quotations from different participant types and across all three country settings, demonstrating cross-country consistency. | **pp. 8–14** |
| 31 | **Clarity of major themes** | Were major themes clearly presented in the findings? | Yes. The Results section is structured around clearly labelled subsections corresponding to: (a) the forces shaping therapeutic navigation (collective decision-making; economic constraints; expectations of cure); (b) three distinct navigation patterns (Pattern 1: Sequential, cost-driven switching; Pattern 2: Concurrent use; Pattern 3: Spiritual-first trajectories in mental health); and (c) concealment as a cross-cutting finding. | **pp. 8–14** |
| 32 | **Clarity of minor themes** | Is there a description of diverse cases or discussion of minor themes? | Yes. Within-pattern variation is described, including: differences in concurrent use driven by economic necessity versus conviction; variation in cost-driven switching between countries with differing insurance coverage; and divergence in mental health trajectories across all three settings regardless of TCIM policy context.  The Discussion addresses minority experiences (e.g. better-resourced patients using concurrent care for spiritual rather than financial reasons) and the limits of Ghana's formal integration model in altering concealment dynamics. | **pp. 11, 15–18** |
