# Supplementary material for: Beyond non-adherence: qualitative insights into how patients strategically navigate pluralistic chronic care in West Africa
Source: BMJ Open. 2026 Oct 15;16(10):e123607. doi: 10.1136/bmjopen-2026-123607 (PMC13691745; doi:10.1136/bmjopen-2026-123607)
Supplement: Supplementary file 3 [file bmjopen-16-10-s002.docx]

Supplementary Table 1. Health system context across study settings

| Indicator | Ghana | Burkina Faso | Niger |
| --- | --- | --- | --- |
| Population (2024) [23] | 34.4 million | 23.5 million | 27 million |
| GDP per capita (USD, 2025) [24] | $2,189 | $952 | $752 |
| Health Insurance Coverage | ~56% (National Health Insurance Scheme, est. 2003). [25] | Limited information, estimates of <10% coverage. [20] | Insurance coverage is among the lowest in West Africa (less than 2%). (40) |
| Out-of-Pocket Health Expenditure [23] | ~25% of total health expenditure | ~35% of total health expenditure | ~40% of total health expenditure |
| Physician Density (per 10,000 population) [26] | 2.66 | 1.48 | 0.38 |
| Health System Structure | 3-tier structure [27]:   1. Primary (Community-Based Health Planning and Services (CHPS) zones/Health posts - Primary Care at the community level. Health centres/clinics and Polyclinic 2. Secondary (District hospitals, Regional hospitals 3. Tertiary (Teaching/specialist hospitals | 4-tier structure [21]:   1. Health and Social Promotion Centres (CSPS) (Primary care at the commune/community level) 2. Medical Centres (CM) and Medical Centres with Surgical Units (CMA) 3. Regional Hospitals (CHR) 4. University Hospitals (CHU) | 3-tier structure [28]:   1. Peripheral Level: Health Posts and Integrated Health Centres (CSI) 2. Regional hospitals (CHR) and Mother and Child Health Centres 3. Central (Tertiary) Level: National (university) hospitals |
| Traditional Medicine Integration [13] | - Ghana has a national policy, dedicated legislation, and a national expert committee for traditional medicine, and its Ministry of Health (MoH) has created specific directorates and offices for traditional and alternative medicine.[14] - Ghana features a robust system for registration and market surveillance of herbal medicines through the Food and Drugs Authority, and several herbal medicines are included in the National Essential Medicines List (NEML). - Ghana leads in research capacity, with institutions such as the Centre for Scientific Research into Plant Medicine and a national pharmacopoeia (the Ghana Herbal Pharmacopoeia). - Traditional medicine training is included at the university level, notably at Kwame Nkrumah University of Science and Technology. - 55 Herbal units formally integrated in various facilities [15] | - Burkina Faso has a well-established national policy on traditional medicine and a legal framework regulating herbal medicines, notably through the “National Policy Framework related to TM and Pharmacopoeia” and relevant public health codes and decrees. - Dedicated administrative structures include a traditional medicine program and national office, as well as a registration system for herbal medicines, with several products listed in the NEML. - The country invests in research, with the Institute for Health Sciences Research and specialised centres active in herbal medicine development. - Traditional medicine is also part of health sciences education curricula. | - Niger has a national policy for traditional medicine, featured in its national health policies and strategies, and has established a directorate and national office within its MoH. - There is a registration system for herbal medicines and inclusion in the national essential medicines list, handled under the Directorate of Pharmacy, Laboratories, and Traditional Medicines. - However, Niger lacks a specialised research institute for traditional medicine, though traditional medicine is included in the curricula of health science studies. - Implementation of this framework is still partial, with weak referral links and limited funding for research into local plants. |
| TCIM Use by Population | ~73-76% of adults report use of herbal medicinal products [29] | Approximately 70–80% of the population uses traditional medicine as a primary health care option [30,31]. | Traditional medicine is the first resort for an estimated 60–80% of the population [32] |
| NCD Burden (% of total deaths as of 2021) [33] | ~46.8% | ~35% | ~24% |
| Hypertension Prevalence (2023) | [dataset] ~35% [34] | [dataset] ~31% [35] | 42% [36] |
| Type 2 Diabetes Prevalence | ~7.5% [37] | ~4.5% [38] | ~4.3% [39] |
